# The effect of music therapy on pain, anxiety and depression in patients after coronary artery bypass grafting

**DOI:** 10.1186/s13019-020-01141-y

**Published:** 2020-05-11

**Authors:** Wang-Sheng Dai, Shu-Ting Huang, Ning Xu, Qiang Chen, Hua Cao

**Affiliations:** 1 Department of Cardiac Surgery, Fujian Maternity and Child Health Hospital, Affiliated Hospital of Fujian Medical University, Fuzhou, China; 2 Department of Cardiovascular Surgery, Union Hospital, Fujian Medical University, Fuzhou, China

**Keywords:** Music therapy, CABG, Pain, Anxiety, Depression

## Abstract

**Objective:**

The purpose of this study was to explore the effects of music therapy on pain, anxiety and depression in patients after coronary artery bypass grafting.

**Methods:**

A retrospective study of 99 patients after coronary artery bypass from January 2017 to January 2019 was conducted in a cardiac center in China. According to the different interventions, all the participants were divided into 3 groups: group A: music therapy; group B: rest without music therapy; and group C: conventional treatment. The Numerical Rating Scale (NRS), Self-Rating Anxiety Scale (SAS) and Self-Rating Depression Scale (SDS) were used to measure the patient’s pain, anxiety and depression before and after 30 min of the intervention.

**Results:**

There were no significant differences in the NRS, SDS and SAS scores between the three groups of patients before the intervention. After 30 min of music therapy, the NRS, SDS and SAS scores of patients in group A were significantly lower than those before music therapy, and the differences were statistically significant. However, before and after the intervention in groups B and C, the NRS, SDS and SAS scores were not statistically significant. By comparison among the three groups after 30 min of intervention, the NRS, SDS and SAS scores in patients in group A were significantly lower than those in groups B and C, and the differences were statistically significant. The scores were not significantly different between groups B and C.

**Conclusion:**

Music therapy can effectively alleviate the pain, anxiety and depression of patients after coronary artery bypass grafting.

## Introduction

Coronary atherosclerotic heart disease is one of the most common heart diseases in elderly individuals. With the development of minimally invasive technology and equipment, many coronary heart diseases can be treated with minimally invasive treatment, including percutaneous coronary intervention, coronary artery bypass grafting with a small left anterior lateral thoracotomy, robotic off-pump coronary artery bypass grafting, etc. However, coronary artery bypass grafting with median thoracotomy is still the main surgical method for the treatment of severe and multiple coronary artery disease [1]. Surgery requires midline splitting of the sternum, a large incision and an indwelling drainage tube; thus, patients usually suffer from obvious postoperative pain [2]. Studies have shown that more than 80% of patients suffer from moderate to severe postoperative pain [3]. Postoperative patients need to be placed in an unfamiliar environment with 24 h of artificial lighting with possible accompanying medical instrument monitoring, no family support, and worries about the success of the operation, all of which can easily make the patients feel anxious and depressed. Although the problems of postoperative pain, anxiety and depression have been taken seriously, the management and treatment are still incomplete [4, 5]. This study summarized our experience in implementing music therapy for patients after coronary artery bypass grafting and evaluated its effects on improving patients’ postoperative pain, anxiety, and depression.

## Information and methods

### Normal information

According to the pain of the three groups of patients in the pilot test, assuming a difference of 10% between the three independent populations, α = 0.05, β = 0.2, it was calculated that 30 participants were required for each group. Assuming a 10% dropout rate, the total sample size required was 99 cases (33 cases per group), which were some of the cases at the hospital at this period. The study was approved by the ethics committee of our university, and written informed consent was obtained from each participant.

This study was a retrospective study conducted by a cardiac hospital. The clinical data of 99 patients who underwent coronary artery bypass graft from January 2017 to January 2019 were collected and divided into 3 groups according to the selected different types of intervention: group A: music therapy group; group B: rest without music therapy group; and group C: conventional treatment group. All of the patients underwent routine surgery with or without cardiopulmonary bypass. The following inclusion criteria were used: tracheal intubation was removed on same the day as the surgery or the next day, hemodynamics were stable, the patient was transferred to the general ward 1 to 2 days after surgery, the analgesia pump was stopped, the patient suffered no hearing impairment, and the patient was able to cooperate with the treatment and this research. The exclusion criteria were as follows: 1, the patient had a history of chronic pain, mental disorders, or hearing impairment, 2, the patient’s condition was combined with other important organ dysfunction, 3, the patient experienced severe complications during the perioperative period, 4, the patient was unable to cooperate with this research or was unwilling to sign the informed consent, and 5, the patient used any painkillers or other methods to reduce pain before our intervention.

## Methods

The patients were lying on their bed in the general ward at 20:00. The surrounding environment was quiet, comfortable, safe, and the light was soft. The bed curtains were pulled closed, and the patients had no interference from the medical staff or researchers. All patients wore binaural headphones. Music was chosen and played in group A for 30 min. Patients could choose their favorite music with the volume based on the their comfort. There was no restriction on the type of music, but mostly light music and relaxing music. The patients had just a quiet rest for 30 min without playing music in group B. The patients in group C were given routine treatment with wearing headphones for 30 min, and normal activities were performed.

### Research tool

Numerical Rating Scale (NRS): This scale has been widely used in pain-related studies with good reliability and validity [6]. The scale uses numbers instead of text to indicate the degree of pain, segmented on a straight line, with the degree of pain expressed with 11 numbers from 0 to 10. 0 means no pain, 1–3 points means mild pain (1 point: no pain in a quiet supine position, pain when turning over and coughing; 2 points: pain when coughing, no pain in deep breathing; 3 points: no pain in a quiet supine, pain with deep breathing and coughing), 4–6 points means moderate pain (4 points: gap pain when in a quiet supine position; 5 points: persistent pain when in a quiet supine position; 6 points: severe pain when in a quiet supine position), 7–10 points means significant pain (7 points: heavier pain, upset, unable to fall asleep; 8 points: persistent, intolerable pain, sweating throughout the body; 9 points: severe, intolerable pain; 10 points: the most painful, life was worse than death).

Self-Rating Depression Scale (SDS): The Zung Self-Rating Depression Scale was used. The SDS has been widely used in clinical practice and has high reliability and validity [7]. The scale consists of 20 items with 4 scoring grades and includes 10 negative symptoms and 10 positive symptoms. Each question represents the characteristics of depression. All of the items reflect mood, physical discomfort, mental activity, behavior and psychological symptoms. According to the frequency of positive symptoms, numbers from 1 to 4 are used for scoring. Based on the frequency of negative symptoms, a rough score is obtained using the reverse score method (4 to 1). The standard score is the score multiplied by 1.25 and is rounded off. The upper limit score is 41, and the standard score is 53. The higher the score, the more pronounced the depression tendency.

Self-Rating Anxiety Scale (SAS): The Zung Self-Rating Anxiety Scale was used. The SAS has been widely used in clinical practice and has high reliability and validity [8]. Fifteen items are expressed as negative words, and scores are based on the frequency of symptoms (1 to 4). Affirmative terms are used to indicate 5 items, and according to the frequency of symptoms, the reverse scoring method (4 to 1) is used for scoring. The scores of all items are summed to obtain the total score. The standard score is multiplied by 1.25 and rounded off. The standard score average is 50. Score description: < 50 means normal, 50 ~ 59 means mild anxiety, 60 ~ 69 means moderate anxiety, and ≥ 70 means severe anxiety.

### Data collection

The SAS, SDS and NRS scores were recorded separately before and after 30 min of the intervention to measure the patient’s pain, anxiety and depression. Participants completed the questionnaire independently; the researchers were not allowed to interfere with the participants’ decisions but were able to explain the questions to the participants or provided language translation. After all questionnaires were completed, the data were statistically analyzed by specialized independent researchers.

### Statistical analysis

Quantitative data are expressed as the means ± standard deviations, and continuous data were compared between groups by analysis of variance; qualitative data were compared between groups by chi-square test; *P* < 0.05 was considered statistically significant.

## Results

The general information of all patients is shown in Table 1. There were no significant differences among the three groups of patients. The NRS, SAS and SDS scores before the intervention were also not statistically significant. These results indicated that the three groups of patients were homogeneous; thus, the data were comparable between groups. After 30 min of music therapy, the NRS, SDS and SAS scores of patients in group A were significantly lower than those before music therapy, and the differences were statistically significant (*P* < 0.05). However, before and after intervention in groups B and C, the NRS, SDS and SAS scores were not statistically significant. After 30 min of intervention, the NRS, SDS, and SAS scores in patients in group A were significantly lower than those in groups B and C, and the differences were statistically significant (*P* < 0.05). The scores were not significantly different between groups B and C. These results indicated that music therapy could significantly reduce the NRS, SDS and SAS scores of patients after coronary artery bypass grafting (Table 2).

**Table 1:** Comparison of general information of the three groups of patients

Variable	Group A	Group B	Group C	*P* value
Age	53.4 ± 12.6	55.8 ± 10.9	53.1 ± 14.8	0.547
Male/female	18/15	17/16	19/14	0.885
Number of bridging vessels				
one	5	4	3	0.932
two	8	7	9	
three or more	20	22	21	
NYHA				
I	0	0	0	0.563
II	23	25	21	
III	10	8	12	
IV	0	0	0	
LVEF	56.9 ± 11.5	58.2 ± 9.3	55.8 ± 12.7	0.612
Incision length	13.1 ± 2.8	14.8 ± 2.1	14.5 ± 2.8	0.886
Marriage				
married	30	31	30	0.906
spinsterhood	1	1	2	
widowed	2	1	1	
Education				
Middle school and below	16	18	15	0.719
High school and technical secondary school	14	10	15	
College and above	3	5	3

**Table 2:** Comparison of NRS, SDS, and SAS scores between the three groups of patients before and after intervention

Scores	Group A	Group B	Group C	P value
NRS				
pretest	7.3 ± 1.9	7.1 ± 1.6	7.2 ± 2.0	0.832
posttest	4.2 ± 2.1*	7.2 ± 1.8^#^	7.4 ± 1.9^#^	0.013
SDS				
pretest	58.6 ± 10.4	60.8 ± 12.8	62.2 ± 13.6	0.761
posttest	46.3 ± 9.3*	59.3 ± 14.1^#^	62.9 ± 11.8^#^	0.022
SAS				
pretest	64.6 ± 13.8	63.2 ± 14.9	62.1 ± 12.7	0.846
posttest	54.3 ± 11.6*	60.5 ± 15.5^#^	62.6 ± 13.9^#^	0.034

# means *P* < 0.05 compared with group A

* means *P* < 0.05 compared with before music therapy

## Discussion

Pain is an unpleasant feeling and emotional experience related to tissue damage or potential tissue damage. It is one of the most common symptoms of surgical patients and affects the recovery of local or overall functions, which can even threaten the patient’s life [9, 10]. Especially for patients with severe coronary heart disease, coronary artery bypass grafting must be performed with a median sternal incision. As the chest cavity moves with breathing and coughing, the cut edges of the sternum will rub against each other, causing severe pain. If the pain cannot be effectively controlled, the patient’s concentrations of catecholamines will increase, which will lead to increased blood pressure, a faster heartbeat, increased peripheral vascular resistance, and increased incidence of postoperative bleeding and will affect the patient’s breathing, cough, mood, and appetite. The corresponding results can delay the patient’s time to get out of bed, affect the patient’s postoperative recovery, and even increase the incidence of complications. Prolonged pain can also easily cause patients to have negative emotions, such as anxiety, anxiety, nervousness and even depression [11, 12]. These effects are more pronounced in older people with weak constitutions who undergo coronary artery bypass grafting. In recent years, increasing amounts of attention have been paid to pain management in these types of older patients. Thus, how to effectively reduce postoperative pain and improve the psychological state of postoperative patients has become a research hotspot.

Although the current treatment of postoperative pain is mainly focused on drug treatment, increasing numbers of nondrug treatment methods have also begun to be widely used [13, 14]. Music therapy is an innovative nondrug treatment that can stimulate the cerebral cortex to reduce stress-related hormones and can have a positive effect on improving emotions through neuroendocrine pathways [15]. It can also have a direct impact on the brain edge and the brainstem reticular structure of the nervous system, reducing sympathetic nervous system activity and increasing parasympathetic neural activity, reducing the body’s physiological response to stress and lowering the cortisol level in the patient’s body, thereby stabilizing the patient’s blood pressure and heart rate under stress [16]. Music therapy relieves pain by distraction and improves the emotional dimension of pain by affecting emotions and feelings. Many domestic and overseas research reports have confirmed that music intervention can have significant effects on pain relief and emotional stability and can relieve stress and anxiety. A clinical randomized controlled trial conducted by Ko SY and his colleagues showed that relaxed music could improve the pain and anxiety of patients undergoing colonoscopy and improve treatment satisfaction [17]. A study by Yaman Aktaş et al. showed that music therapy could be used as a nondrug therapy to reduce pain in patients with mechanical ventilation [18]. Ernsten and his teams found that perioperative music therapy could effectively relieve acute pain associated with surgery [19]. Rohilla et al. showed that music therapy could help reduce anxiety, pain and the use of opioids during surgical dressing changes in burn patients [20]. Music therapy can relieve the degree of postoperative pain in surgical patients and can be beneficial in stabilizing the patient’s heart rhythm and maintaining the hemodynamic balance, as shown in a study by Chen and her colleagues [21]. Music can adjust and balance people’s emotions. It can slow down the heart rate and dilate the blood vessels, thereby improving the blood circulation, reducing the heart load, removing the metabolic products produced by myocardial hypoxia, and consequently alleviating angina pectoris [22]. In this study, the pain score of patients in the music therapy group was significantly lower than that of patients who did not receive music therapy and that of the preintervention status; this study also confirmed that music therapy could relieve pain in patients after coronary artery bypass grafting.

In addition, music also had a good sedative effect [23, 24]. Music stimulated the right hemisphere limbic system, which manages emotions, feelings and sensory centers to bring about a sedation effect and improve the patient’s negative emotions. When patients listen to comfortable and sweet-sounding music, they produce pleasant associations, which can create a comfortable and satisfied mood to relieve anxiety. This study also showed that the SAS and SDS scores of the music therapy group after music therapy were significantly lower than those before the music therapy, and the SAS and SDS scores of the music therapy group were significantly lower than those of the non-music therapy group. Such results indicated that listening to comfortable and pleasant music could significantly relieve the patients’ anxiety and depression.

The study still had some shortcomings. First, this was a single-center retrospective study, not a randomized controlled study; thus, there was a certain deviation in the selection of cases, but the findings still had certain clinical significance. In addition, this was a single-center study with a relatively small sample size. A multicenter and large-sample study will be performed in the future. Third, this study was only limited to specific patients undergoing coronary artery bypass surgery. Whether music therapy had the same effect in other patients undergoing other types of surgery will require further study.

## Conclusion

Music therapy as a nondrug treatment method is very safe, simple and economical to implement and can effectively alleviate the pain, anxiety and depression of patients undergoing coronary artery bypass grafting. Thus, it is worthy of clinical promotion and application.

## Data Availability

Data sharing not applicable to this article as no data sets were generated or analyzed during the current study.

## Abbreviations

NRS: Numerical Rating Scale
SAS: Self-Rating Anxiety Scale
SDS: Self-Rating Depression Scale
